# CSAC-Net: Fast Adaptive sEMG Recognition through Attention Convolution Network and Model-Agnostic Meta-Learning

**DOI:** 10.3390/s22103661

**Published:** 2022-05-11

**Authors:** Xinchen Fan, Lancheng Zou, Ziwu Liu, Yanru He, Lian Zou, Ruan Chi

**Affiliations:** 1 Electronic Information School, Wuhan University, Wuhan 430072, China; fanxinchen@whu.edu.cn (X.F.); zoulancheng@whu.edu.cn (L.Z.); liuziwu@whu.edu.cn (Z.L.); heyanru@whu.edu.cn (Y.H.); 2 Hubei Three Gorges Laboratory, Yichang 443007, China; rac@wit.edu.cn

**Keywords:** surface electromyography, gesture recognition, meta-learning, attention convolution network, short time Fourier transform

## Abstract

Gesture recognition through surface electromyography (sEMG) provides a new method for the control algorithm of bionic limbs, which is a promising technology in the field of human–computer interaction. However, subject specificity of sEMG along with the offset of the electrode makes it challenging to develop a model that can quickly adapt to new subjects. In view of this, we introduce a new deep neural network called CSAC-Net. Firstly, we extract the time-frequency feature from the raw signal, which contains rich information. Secondly, we design a convolutional neural network supplemented by an attention mechanism for further feature extraction. Additionally, we propose to utilize model-agnostic meta-learning to adapt to new subjects and this learning strategy achieves better results than the state-of-the-art methods. By the basic experiment on CapgMyo and three ablation studies, we demonstrate the advancement of CSAC-Net.

## 1. Introduction

Surface electromyography (sEMG) is a superimposed electrical signal formed on the surface of human skin, by the motor unit action potential (MUAP) of motion related muscles propagating along the direction of muscle fibers. This is an important human biological resource, which contains a wealth of information associated with one’s movement. Compared with the conventional control method based on programs, sEMG-based algorithms can better reflect the intention of human movement, and this can be used in bionic prosthetics, exoskeleton robots, medical rehabilitation robots, and so on [1,2]. For example, in the field of virtual reality, Nadia Nasri et al. proposed a sEMG-controlled 3D game that included a deep learning-based architecture for real-time gesture recognition in [3], which allows amputees to use low-cost sEMG sensors to play the game. Thus, there is a natural advantage in utilizing sEMG signals as interface media to construct human–robot interaction (HRI) systems [4,5]. In order to realize such function, the basic task is gesture recognition.

However, the quality of information decoded from sEMG can differ, largely depending on the processing techniques. As an example, a motion intention detection (MID) problem involving shoulder muscle contractions was assessed in [6]. Andrea Tigrini et al. considered multiple aspects to capture the best characteristics of the movement, including signals segmentation, feature extraction, and classification. Therefore, a large number of studies focus on processing techniques—using different features and classifiers to improve the classification accuracy on specific sEMG datasets. Previously, many traditional machine learning methods are used for this task, including Linear Discriminant Analysis (LDA) [7], Support Vector Machine (SVM) [8], Bayesian classifier, and Gaussian Mixture Model (GMM) [9]. In recent years, deep learning’s strong fitting ability has made it popular to use an artificial neural network for sEMG classification. In [10], Tsinganos et al. proposed using Temporal convolutional network (TCN) in gesture recognition, yielding an improvement of almost 5% to the state of the art reported in the literature. In [11], Chen et al. proposed a compact convolutional neural network (CNN) to achieve excellent performance with fewer parameters. Even though the current work can realize high prediction accuracy on specific datasets in gesture recognition through sEMG, when we apply such technology to human–robot interaction, there are still several challenges. (1) sEMG gesture recognition is difficult to support by large datasets for daily use and promotion. At the same time, due to electrode offset and individual differences, if only ordinary machine learning or deep learning technology is utilized to deal with gesture recognition of new users without any designed learning strategy or specific network design, the obtained model will face serious overfitting [12,13]. (2) Previous networks such as RNN and LSTM have poor efficiency and need a lot of time to train new samples. In other words, it takes a lot of time to update the model. Therefore, it is difficult to ensure these algorithms can be put into practice quickly [14]. (3) There is no unified standard for the processing of raw data. Experiments show that different preprocessing schemes and different feature representations have great influence on the results [4,15].

In this article, we propose a novel framework for gesture recognition, called Channel-Spatial Attention Convolution Network (CSAC-Net). In CSAC-Net, we design a convolutional network supplemented by a special attention mechanism [16], with which the multi-channel time-frequency information can be extracted effectively. For fast adaption, we utilize the Model Agnostic Meta-Learning (MAML) [17] algorithm, which enables our CSAC-Net to update parameters quickly and effectively with numerous different learning tasks and achieves better classification performance for new users and gestures compared with previous work. In summary, the main contributions and novelties are listed below:

- To mitigate the effect of individual differences, CSAC-Net is used to update the model based on MAML. Compared with the traditional learning method, it focuses on improving the overall learning ability of the model, rather than the ability to solve a specific sEMG classification problem.
- To meet the challenge of model complexity and feature extraction, we combine the lite CNN network with the attention mechanism. By analyzing the spectrogram in the time-frequency domain of multi-channel sEMG signal after preprocessing, the features of sEMG signal are effectively extracted, which contributes to better performance than utilizing raw sEMG data in the time domain.
- CSAC-Net requires the maximization of the sensitivity of loss functions of new tasks with respect to the parameters when we are training the model. Minimal parameter changes can bring great improvement to the model. In this way, it is possible to quickly adapt to new tasks with only a small amount of data by gradient adjustment on the basis of initial parameters.
- In order to demonstrate the generalization and fast adaptation of the classification of our model, three datasets are selected to carry out the experiments. Our model achieves better performance than previous work.

## 2. Related Works

### 2.1. Traditional Methods for Gesture Recognition through sEMG

In general, gesture recognition based on collected sEMG has two inevitable processes: feature extraction and classification model.

For feature extraction, there are time-domain features, frequency domain features, and time-frequency-fusion features. Appropriate sEMG feature combination can effectively improve the accuracy of gesture recognition [18]. In [19], Triwiyanto et al. utilized root mean square (RMS), variance (VAR), mean absolute value (MAV), and simple square integral (SSI) to analyze the EMG signals in the embedded platform. The four time-domain features require less computational force; thus, they are vital to ensure real-time processing. In [20], Leserri et al. made use of the mean of signal frequencies (MNF) calculated with FFT, claiming that frequency domain features potentially yield relevant information for movement prediction. For the classification model, support vector machine (SVM) [8], *K*-nearest neighbour (KNN) [19], and some other classic algorithms were used frequently in gesture recognition. In [21], the authors used LDA to explore the impact of varying the number of electrodes and segmentation window sizes on EMG decoding accuracy, as it is the most commonly used for the classification of limb movements [22]. Hancong et al. also utilized LDA for a low-power embedded system in [23], in that LDA features low requirements for computing power. Moreover, with the rise of deep learning, it gradually constitutes an element of edge research. In [24], a new deep, one-dimensional CNN model was proposed to classify six types of hand movements, obtaining the highest accuracy of 94.94%. Xin et al. proposed combining the advantages of PCA and neural network and make adjustments by scale unscented Kalman filter [25], improving the accuracy and reliability of the lower limb motion classification.

The methods shown above mainly focus on improving the accuracy of sEMG classification in specific datasets. When it is put into practical application in real life, sEMG will change with people’s physical signs (including gender, age, limb health, disability, etc.) due to its subject specificity [5]. This leads to the trained model easily failing, lowering the accuracy and stability. Therefore, new methods are necessary.

### 2.2. Deployment of Attention Mechanism in Biomedical Signal Decoding

As the attention mechanism enables the model to focus on the most important part of the input sequences [26], it is widely used to reduce the complexity of the model. In [27], Tao Wei et al. proposed using channel-wise attention to optimize the EEG-based emotion recognition, because it can change the weight of different channels to explore more effective information in a feature map. Additionally, channel-wise attention can be easily integrated into CNN architectures [28]. Another important kind of attention is spatial attention. Guoqi Chen et al. added spatial attention after reducing pooling, retaining a lot of ancillary information and finding out critical information quickly [29], with which their TDACAPS model achieved the state-of-the-art result. Moreover, Yongqing et al. combined Channel Attention and Spatial Attention to process the sEMG signal, providing new ideas for muscle fatigue detection [30].

In summary, the attention mechanism can evaluate the importance of different features, both on a spatial and channel level. Thus, the model can obtain better prediction ability and low complexity. Because sEMG is usually a multi-channel sequence, Channel-Spatial Attention cannot be more appropriate to improve the performance of the model.

### 2.3. Current Progress in Domain Adaptation

In view of the differences caused by the subject specification of sEMG and the electrodes used to collect neuroelectricity, much work has been done to mitigate the impact. In 2017, Du et al. embedded a deep domain adaptation mechanism into the gesture classifier. The adaptation starts working after the device is worn. In spite of this, the accuracy of the inter-subject with adaptation can only reach 35.1% [31]. In [32], Guangjun et al. proposed a feature extraction method based on the gray model to find the connection between different kinds of subjects. However, this requires high consistency of the physical characteristics of the electrodes and the statistical distribution of the signal. Rahimian et al. proposed using few-shot learning to adapt to new users [33]. Although high accuracy (76.39%) was achieved in the inter-subject experiment, the feature made use of was only the raw sEMG. In other words, much hidden information is not effectively mined and utilized. Moreover, Banluesombatkul et al. carried out the first work that investigated MAML, a non-conventional pre-training method [34]. This article demonstrates that MAML is possible for electroencephalography (EEG) classification.

In summary, the methods listed have achieved substantial success, but there are still a few limitations. For one thing, the optimization of new subjects is limited to specific backgrounds and therefore has a lack of universality. Furthermore, the concept of meta-learning has not received enough attention. There is still much room for improvement. In fact, learning from the experience that human beings can quickly learn new knowledge through only a few samples, meta-learning can make the model learn to learn.

## 3. Methods and Theories

**Figure 1:**
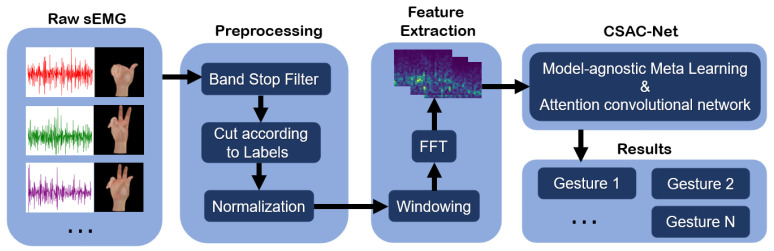
An overview of the method in a block diagram representation.

An overview of the method in a block diagram representation is shown in Figure 1. In this section, we will introduce all the procedures in detail.

### 3.1. Data Preprocessing

In order to obtain the sEMG with less interference, preprocessing is of vital importance. It usually includes filtering, smoothing, and normalization [31,35,36]. Following [31], we remove the power-line interference with a 45–55 Hz, second-order Butterworth filter. Then, the middle one-second window is used to cut out sEMG with the main information. Additionally, we normalize the data, reducing the influence of singular samples on the results. After proprocessing, we obtain the standard dataset where a single sample is organized as a one-dimensional vector with the length of 1000.

### 3.2. Feature Extraction

The chief feature we utilize is short-time Fourier transform (STFT). It is a kind of signal transform related to Fourier transform. The basic idea is to add a sliding time window function to the signal and carry on the Fourier transform to the signal in the window to obtain the time-varying spectrum of the signal. Mathematically, the continuous form and discrete form are written as:

(1) $$STFT\left\{x\left(t\right)\right\}\left(\tau ,\omega \right)=\int_{-\infty }^{\infty }x\left(t\right)w\left(t-\tau \right)e^{-i\omega t}dt$$

(2) $$STFT\left\{x\left[n\right]\right\}\left(m,\omega \right)=\sum_{n=-\infty }^{\infty }x\left[n\right]w\left[n-m\right]e^{-j\omega n}$$

where w(τ)/w(m) represents a window function, including Hann window, Hamming window, etc. Furthermore, x(t)/x[n] is the sEMG signal waiting to be processed.

**Figure 2:**
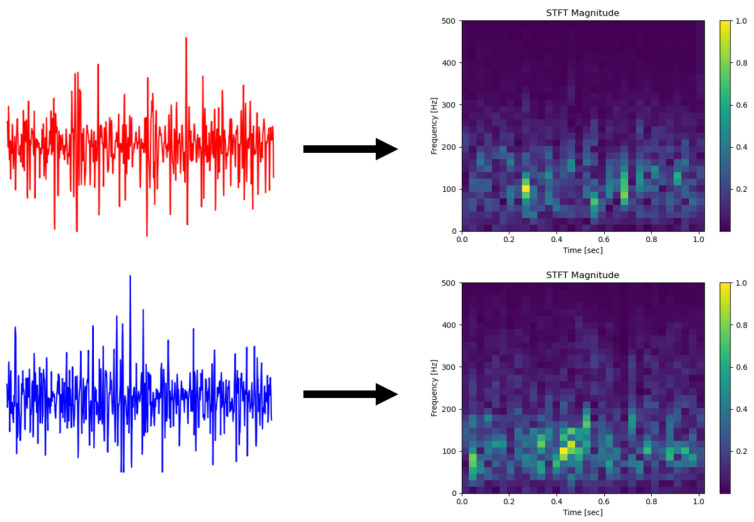
Two processed surface EMG samples and their STFT spectrogram (Hanning window, window length = 64).

In the application of STFT, the length of the window determines the time resolution and frequency resolution of the spectrogram, so it is necessary to make a trade-off in practice. When processing the CapgMyo dataset, we select the Hanning window and the window length is 64. After STFT, we obtain the time-frequency images of processed sEMG, whose size is 33×33. The results are shown in Figure 2. As is vividly shown, the law of frequency characteristics changing with time can be captured.

### 3.3. The Architecture of CSAC-Net

**Figure 3:**
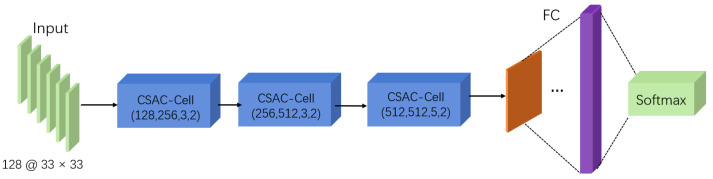
The detailed architecture of the proposed CSAC-Net.

The detailed architecture of the proposed CSAC-Net is shown in Figure 3. The designed network architecture is mainly composed of a convolutional neural network with a novel Channel-Spatial Attention Mechanism. The Channel-Spatial Attention Mechanism combines the advantages of Channel Attention and Spatial Attention, which can adaptively select both important objects and regions [37]. Channel-Spatial Attention is suitable for our input form of a 33×33 feature map image with 128 channels to extract important information. The proposed CSAC-Net can be regarded as being composed of three Channel-Spatial Attention Convolution cells (CSAC-cells), one fully connected layer, and Softmax. The Channel-Spatial Attention Module, CSAC-Cell, and loss function are described in detail below.

#### 3.3.1. Channel-Spatial Attention Module

**Figure 4:**
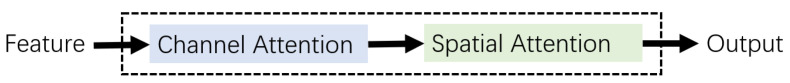
The Channel-Spatial Attention Module.

As shown in Figure 4, The Channel-Spatial Attention Module (CSAM) consists of one Channel Attention Module and one Spatial Attention Module. The model notices unimportant features through an attention mechanism and sets them to zero through the soft threshold function; in other words, we can notice important features through the attention mechanism and keep them. The details of the Channel Attention Module and Spatial Attention Module are described, respectively, in the following.

**Figure 5:**
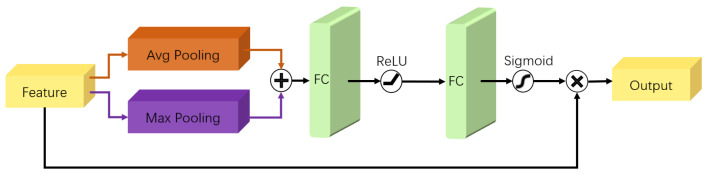
The structure of Channel Attention Module.

As shown in Figure 5, the Channel Attention Module (CAM) is divided into two parts: the compression module and the excitation module. The compression module collects global spatial information by means of the sum of global average pooling (GAP) and global max pooling (GMP). The excitation module captures the relationships on the channels and outputs an attention vector by using ReLU and Sigmoid. Each channel of the input feature is then scaled by multiplying the corresponding elements in the attention vector. In summary, the Channel Attention Module $F_{CAM}$ (with parameter θ), with *X* as the input and *Y* as the output, can be formulated as:

(3) $$Y=F_{CAM}\left(X,\theta \right)=X\sigma \left(W_{2}\delta \left(W_{1}\left(GAP+GMP\right)\right)\right)$$

**Figure 6:**
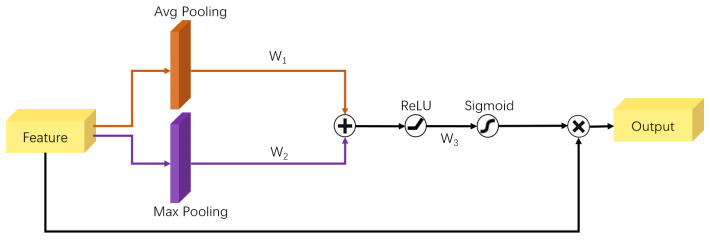
The structure of Spatial Attention Module.

As shown in Figure 6, the Spatial Attention Module (SAM) is also divided into two parts: the compression module and the excitation module. The compression module collects global channel information by means of the sum of global average pooling and global max pooling with parameters. The excitation module captures the spatial relationships and outputs an attention vector by using ReLU and Sigmoid. The input feature is then scaled by multiplying the corresponding elements in the attention vector. In summary, the Spatial Attention Module $F_{SAM}$(with parameter θ), with *X* as the input and *Y* as the output, can be formulated as:

(4) $$Y=F_{SAM}\left(X,\theta \right)=X\sigma \left(W_{3}\delta \left(W_{1}GAP+W_{2}GMP\right)\right))$$

#### 3.3.2. CSAC-Cell

**Figure 7:**
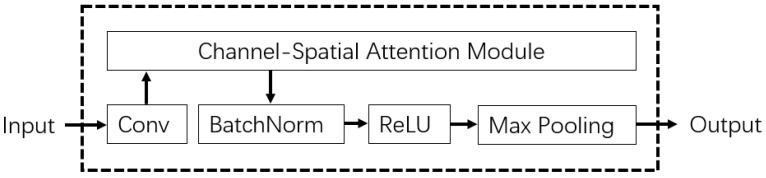
The structure of the CSAC-Cell.

As shown in Figure 7, the CSAC-Cell consists of one Conv2D layer with input channel size $C_{1}$, output channel size $C_{2}$, and kernel size $K_{1}$, one Channel-Spatial Attention Module (CSAM), BatchNorm, ReLU, and Maxpool2D with kernel size, and padding $K_{2}$.

#### 3.3.3. Loss Function

The proposed CSAC-Net uses the cross-entropy loss function as the loss function, which can be formulated as:

(5) $$Loss=-\sum_{i=1}^{n}y_{i}\log\left(\hat{y_{i}}\right)$$

where *n* represents the number of the hand gesture categories, $y_{i}$ represents the truth value of the *i*th category, and $\hat{y}$ represents the predicted value of the *i*th category of the output. The gradient of the weight of the loss function for the last layer is no longer related to the derivative of the activation function but only proportional to the difference between the output value and the real value. At this time, the convergence is faster and the back-propagation has even multiplication, so the update of the whole weight matrix will be accelerated.

### 3.4. Evaluating Indicator: *N*-Way *K*-Shot

For meta-learning tasks, the commonly used evaluation method is called *N*-way, *K*-shot [38]. Generally, N∈{5,10,15,20},K∈{1,5}. During the training stage, *N* × *K* samples are selected for training. In the verification phase and test phase, *K* samples are selected from *N*-class samples to perform the *N*-way *K*-shot classification task. In fact, it is a sampling of the original dataset. The predicted category is determined according to the results. Finally, the accuracy rate of the predicted category matching the actual category is the evaluation indicator.

### 3.5. MAML Framework

In deep learning, a common skill to overcome the defects of training data is pre-train-fine tune. That is, pre-train the model on the large dataset, and then fine tune the weight on the small dataset. However, when the training data are extremely scarce, this technique cannot work. Furthermore, sometimes this method will make the model fall into local optimal solution. Thus, to mitigate the effect of individual differences and gain faster adaptation without overfitting, MAML is used to train models.

**Figure 8:**
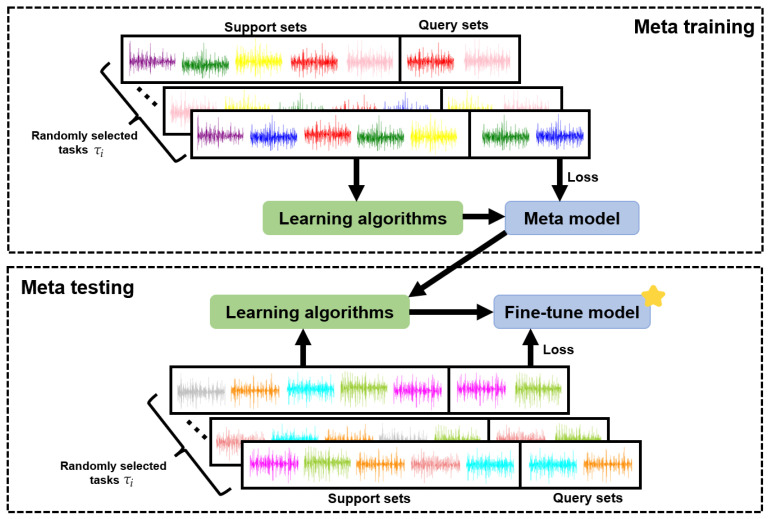
Specific block diagram of MAML framework in our work. The colorful lines represent the sEMG signals used for training and verification after windowing. The yellow star represents the model for new subjects.

This method initializes model parameters for different task scenarios by meta training through gradient descent in the meta-training stage, and in the meta-testing stage, meta testing can achieve good identification results by fine tuning in several steps. This is shown in Figure 8.

The basic processing unit of meta-learning is a task. We formally introduce the structure as follows. Each task $T=\left\{L\left(x_{1},a_{1},\ldots ,x_{H},a_{H}\right),q\left(x_{1}\right),q\left(x_{t+1}|x_{t},a_{t}\right),H\right\}$ consists of a loss function L, a distribution over initial observations $q\left(x_{1}\right)$, a transition distribution $q\left(x_{t+1}|x_{t},a_{t}\right)$, and an episode length H. One task consists of a support set and a query set. The total number of support set samples is $NK=Num\left|\left\{x_{ij}|1⩽i⩽N,1⩽j⩽K\right\}\right|$ and the total number of query set samples is $M=Num\left|\left\{x_{l}^{\prime }|1⩽l⩽M\right\}\right|$. In general, the support set and query set in each task follow two principles of non-intersection: (1) There is no crossover between the support set sample and the query set sample. (2) There is crossover between support set categories and query set categories. That is:

(6) $$\left\{x_{ij}|1⩽i⩽N,1⩽j⩽K\right\}\cap \left\{x_{l}^{\prime }|1⩽l⩽M\right\}=\Phi$$

(7) $$\left\{C_{x_{ij}}|1⩽i⩽N,1⩽j⩽K\right\}\cap \left\{C_{x^{\prime }}|1⩽l⩽M\right\}=\Phi$$

Therefore, the key of MAML is the acquisition of initial parameters under the new task, namely meta training. This stage is completed in two steps: Base-learner learning stage, mainly learning the attributes of specific tasks; Meta-learner learning stage, mainly learning the commonness of different tasks. We take an explicit approach to this problem: because the model will be fine tuned using a gradient-based learning rule on a new task, we aim to train a model in such a way that this gradient-based learning rule makes rapid progress on new tasks drawn from the task without overfitting. According to [17], we conclude the pseudo code of the Algorithms 1 and 2 as follows.

**Algorithm 1** MAML-training**Require:** p(T): distribution over tasks **Require:** α, β: step size hyperparameters **Require:** Iteration number of epoch model 1: randomly initialize θ 2: **while** not epoch **do** 3:   Sample batch of tasks $T_{i}∼p\left(T\right)$ 4:   **for all $T_{i}$ do** 5:     Sample K datapoints $D=\left\{x^{\left(j\right)},y^{\left(j\right)}\right\}$ from $T_{i}$ 6:     Evaluate $\nabla_{\theta }L_{T_{i_{\mathrm{support}\;}}}\left(f\left(\theta \right)\right)$ with respect to ^NK^ examples 7:     Evaluate $\theta_{i}^{\prime }=\theta -\alpha \nabla_{\theta }L_{T_{i_{\mathrm{support}\;}}}\left(f\left(\theta \right)\right)$ 8:     Evaluate $\sum_{i}L_{T_{i_{\mathrm{support}\;}}}\left(f\left(\theta_{i}^{\prime }\right)\right)$ 9:   **end for** 10:  Update $\theta =\theta -\beta \nabla_{\theta }\sum_{T_{i}∼p\left(T\right)}L_{T_{i_{\mathrm{support}\;}}}\left(f\left(\theta_{i}^{\prime }\right)\right)$ 11: **end while**

**Algorithm 2** MAML-testing**Require:** training data $D_{T}^{\mathrm{tr}}$ new task *T* **Require:** learned θ 1: Evaluate $\nabla_{\theta }L\left(\theta ,D_{T}^{\mathrm{tr}}\right)$ 2: Compute adapted parameters with gradient descent $\varphi_{i}=\theta -\alpha \nabla_{\theta }L\left(\theta ,D_{T}^{\mathrm{tr}}\right)$

## 4. Experiments and Results

In this section, we first introduce the datasets used to evaluate the proposed CSAC-Net. Then, we analyze several experiments we performed and the corresponding results in detail.

The model was developed in Python using Pytorch and learn2learn [39] libraries. The training was performed on a Desktop PC with an Intel i5-12400F 16-Core Processor, 16 GB RAM, and an Nvidia GeForce RTX 3060 12GB GPU.

### 4.1. Dataset

In the experiments, the CapgMyo [31] dataset was used to evaluate our proposed CSAC-Net. The CapgMyo dataset includes HD-sEMG data of 128 channels acquired from 23 intact subjects. The acquisition device has a matrix-type (8 × 16) differential electrodes array with silver wet electrodes. Each gesture in the CapyMyo is held for 3–10 s and repeated 10 times and there is a resting posture lasting 7 s between each movement. The CapgMyo dataset consists of 3 sub-datasets (DB-a, DB-b, and DB-c). Until now, many researches have achieved good results (which are even saturated) on supervised learning [40,41,42]. However, the task of inter-subject gesture recognition still has much room for improvement [31,43].

### 4.2. Basic Experiments

***Experiment 1—Classification with Different Input Forms by Supervised Learning***

In this part, we performed three experiments of traditional supervised learning on DB-a, DB-b, and DB-c, respectively. In the three training groups, the raw sEMG signal, the FFT spectrum, and the STFT spectrogram after data preprocessing were used as input, respectively. Both types of frequency domain input can be thought of as images. The raw sEMG signal is a 1000 × 128 size image with 1 channel, the FFT spectrum is an image with 1 channel, and the STFT spectrogram is a 33 × 33 size image with 128 channels.

The initial learning rate was 0.001, the number of training epochs was 300, and the batch size was 64. After 150 epochs, the learning rate was set to 0.0001. Adam was utilized as the optimizer with the weight decay set to 0.0001 and the cross-entropy loss function used as loss function. The ratio of the training set, validation set, and test set of the three datasets was 6:2:2.

From Table 1, it can be observed that the STFT spectrogram in the time-frequency domain performs much better than the raw sEMG in the time domain and the FFT spectrum in the frequency domain using the proposed CSAC-Net. We perceive that this is because the STFT spectrogram contains richer feature information and that our well-designed CSAC-Net is more suitable for multi-channel images. Utilizing CSAC-Net for supervised learning with STFT spectrogram as input, the accuracy can achieve approximately 94% on the DB-a, DB-b, and DB-c.

Apart from that, we selected some baselines to make comparisons. As is shown in Table 2, Resnet18 is trained with the same hyperparameters as CSAC-Net. For ED-TCN and other traditional methods, mean average value (MAV) is a more suitable classification feature than STFT Spectrogram according to [44]. MAV is defined as:

(8) $$\mathrm{MAV}=\frac{1}{N}\sum_{i=1}^{N}\left|x_{i}\right|$$

where N represents the window length. When we computed MAV of sEMG, the window length was set to 200 ms and the sliding step was 25 ms. Finally, we took the average accuracy of 10 times as the final result, which was shown in Table 2.

From Table 2, compared with previous classification methods (both sequential models and frame-wise models), the proposed CSAC-Net achieved better performance in the traditional supervised learning task.

***Experiment 2—Classification on New subject with MAML***

In this experiment, we demonstrated that the proposed CSAC-Net can accurately recognize the gestures on new subjects by training only a small number of samples with an STFT spectrogram used for input. We divided DB-a, DB-b, and DB-c into meta-train dataset ($D_{meta-train}$), meta-validation dataset ($D_{meta-val}$), and meta-test dataset ($D_{meta-test}$), respectively. The same subject did not exist in different metasets. There was no overlap between the metasets. For DB-a, $D_{meta-train}$ consisted of the first 16 subjects and $D_{meta-val}$ included the 17th subject’s data. Furthermore, we used the 18th subject for $D_{meta-test}$ to evaluate our model. For DB-b, $D_{meta-train}$ consisted of the first 8 subjects and $D_{meta-val}$ included the 9th subject’s data. In addition, we used the 10th subject for $D_{meta-test}$ to evaluate our model. Furthermore, for DB-c, $D_{meta-train}$ consisted of the first 6 subjects and $D_{meta-val}$ included the 7th subject’s data and we used the 8th subject for $D_{meta-test}$ to evaluate our model.

The Adam optimizer was used with the learning rate (meta-learning rate) set to 0.001. The loss function was cross-entropy function. Fast learning rate was set to 0.1, which was used in MAML with the learn2learn framework. For the *N*-way *K*-shot, we carried out 5-way 1-shot and 5-way 5-shot in this experiment with different meta batch size. A large number of tasks were constructed by random sampling from each metadata set, which was more practical. Each task consisted of *N*-way and 2 × *K*-shot, half of which was for training and half was for verification. The number of tasks in each metaset was set to 20,000. The iteration was set to 1. There are two reasons for iterating only once: (1) With few shot data, too many updates will lead to over fitting. (2) The model needs to be efficient, that is, we can quickly find the best parameters through only one update. When testing the model, we can iterate more than once until we obtain the best results.

From Table 3 and Table 4, we can determine that the classification accuracy is related to the meta batch size. Overall, generalization and adaptability are better when meta batch size is set to 8. In [31], the inter-subject with adaptation classification accuracy was 55.3% for DB-b, and the inter-subject with adaptation classification accuracy was 35.1% for DB-c. The results in Table 3 and Table 4 show that CSAC-Net performed particularly well in terms of adaptability to the new subject.

**Figure 9:**
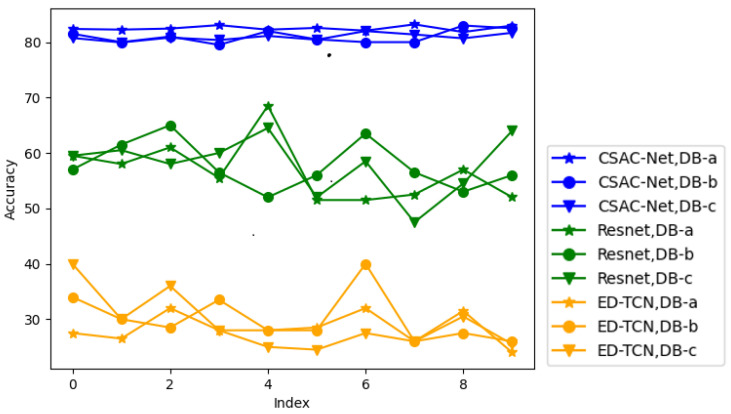
For different methods and datasets, the accuracy of 10 repetitions.

As shown in Table 5, we compared the performance of the hand gesture classification on a new subject between our model and previous methods. Du et al. adopted the method of Deep Domain Adaption for hand gesture classification on a new subject. They obtained the accuracies of 55.3% and 35.1% on DB-b and DB-c, respectively. Sibasankar et al. proposed a method of multilinear singular value decomposition and dictionary learning (MLSVD + DL) for hand gesture classification on a new subject and achieved the accuracies of 75.4% and 68.3% on DB-b and DB-c, respectively. Furthermore, CSAC-Net with MAML obtains the accuracies of 82.50%, 81.00%, and 80.91% on DB-a, DB-b, and DB-c. Moreover, we implemented Resnet18 and ED-TCN (referred to above) with MAML to make a comparison with our network. In order to illustrate the real classification ability of different methods, we calculated the mean accuracy of these architectures. It can be seen that the proposed CSAC-Net with MAML has excellent performance for hand gesture classification on a new subject. Meanwhile, as is illustrated in Figure 9, the stability of the proposed CSAC-Net was better than other methods, with the curve showing little jitter.

### 4.3. Ablation Studies

***Experiment 1—Classification of Different Input Forms on New Subject with MAML***

To evaluate the importance of feature selection in our proposed CSAC-Net, in this ablation study, we changed the input of basic experiment 2 into the raw sEMG signal and the spectrum obtained by FFT. Then, we observed the gesture recognition effect of these two inputs on a new subject and compared them with the results of basic experiment 2. The hyperparameters of the model were consistent with those of the basic experiment 2.

As can be seen from the results in Table 6 and Table 7, the FFT spectrum as input is slightly better than the raw sEMG signals. However, compared with the results in Table 3 and Table 4, both of them are far inferior to the STFT spectrogram in carrying out the inter-subject gesture classification task. We can safely conclude that the STFT spectrogram greatly improves the gesture recognition performance of CSAC-Net for a new subject.

***Experiment 2—Classification on New Subject with Pretrained Model***

To evaluate the importance of MAML in CSAC-Net when classifying the gestures of new subjects, in this ablation study, we tested a new subject using a pretrained model. The method of pretraining by supervised learning is similar to that of basic experiment 1. The hyperparameters were consistent with basic experiment 1. For DB-a, first 16 subjects were used for training (70%) and validation (30%), and the remaining 2 subjects for testing. For DB-b, the first 9 subjects (ID: 1–18, including 2 sessions) were used for training (70%) and validation (30%), and the remaining 1 subject (ID: 19 and 20) was used for testing. Furthermore, for DB-c, the first 8 subjects were used for training (70%) and validation (30%), and the remaining 1 subject was used for testing.

It can be seen from Table 8 that although the pretrained model has high accuracies in the validation sets, it shows a poor performance in the test sets. That is, when the pre-trained recognition model is applied to new users, individual differences, such as differences in posture information, muscle contraction mode, and contractile force, will make the model fail. At the same time, pretraining takes a lot of time. Comparing the results in Table 1 and Table 9 with the results in Table 8, we can see the superior performance of CSAC-Net. Not only does it show outstanding adaptability to a new subject, but it also requires very little training time. Hence, MAML greatly improves the learning efficiency of our proposed fast adaptive CSAC-Net for a new subject.

***Experiment 3—Classification on New Subject with Different Models***

In this ablation study, we tried to use three different networks to train CapgMyo in order to illustrate the merits of CSAC-Net on feature extraction for a new subject. The training method and the setting of hyperparameters were the same as those in basic experiment 2, except for some changes in the network model. For SAC-Net, we removed the Channel Attention Mechanism in CSAC-Net and replaced it with a conventional convolution layer to keep the overall complexity of the model unchanged. For CAC-Net, we removed the spatial attention mechanism in CAC-Net and replaced it with conventional convolution layer. Furthermore, for CNN, we removed the Channel-Spatial Attention Mechanism in CSAC-Net and replaced it with the conventional convolution layer.

According to the results in Table 10 and Table 11, meta batch size also has an impact on the classification accuracy of the model. Although some models can achieve quite good classification performance in some specific databases for 5-way 5-shot classification, they are generally weaker than CSAC-Net in terms of generalization and adaptability. Therefore, the CSAC-Net network architecture also plays an important role in gesture classification on a new subject.

## 5. Conclusions

In this article, we have proposed a novel learning strategy for sEMG gesture recognition, namely CSAC-Net. CSAC-Net can overcome the difficulty caused by subject specificity of sEMG, being fast and adaptive. The mean accuracy of inter-subject gesture recognition on the CapgMyo dataset achieves 82.50%, 81.00%, and 80.91% (DB-a, DB-b, and DB-c), exceeding the state-of-the-art method. To realize this, one important factor is the MAML learning framework, which enables the parameters of the model to update rapidly when we deal with new subjects. Meanwhile, the STFT spectrogram combined with a well designed convolutional network lays a solid foundation for the feature extraction and classification ability of the model. Channel-Spatial Attention helps to keep the focus on important information when there are many channels and feature maps. In the traditional supervised learning experiment, our network achieves 94.10%, 94.06%, and 94.44% on DB-a, DB-b, and DB-c, respectively. The ablation studies also reveal that each part of the model plays an important role.

In the future, we will devote effort to optimizing the CSAC-Net and developing a hardware system to implement our model, which will demonstrate that CSAC-Net possesses a good application prospect in the field of human–computer interaction in practice.

## Figures and Tables

**Table 1:** Traditional supervised learning classification accuracy (%) of different input forms on CapgMyo (18 subjects and 8 gestures for DB-a, 10 subjects and 8 gestures for DB-b, 10 subjects and 12 gestures for DB-c). The boldface numbers represent the best classification accuracies for each dataset.

Input	DB-a	DB-b	DB-c
Raw sEMG	46.18	52.81	42.59
FFT Spectrum	67.71	73.13	67.13
STFT Spectrogram	**94.10**	**94.06**	**94.44**

**Table 2:** Comparison between previous methods and the proposed CSAC-Net in terms of the accuracy (%) of traditional supervised learning classification on DB-a, DB-b, and DB-c (10 times average accuracy). The boldface number represents the best classification results.

Methods	DB-a	DB-b	DB-c
**STFT Spectrogram + CSAC-Net**	**94.10**	**94.06**	**94.44**
STFT Spectrogram + Resnet18 [45]	91.32	87.81	89.81
MAV + ED-TCN [44]	93.75	91.88	90.28
MAV + KNN (k = 3) [46]	88.54	88.75	91.67
MAV + SVM ( kernal: Gaussian ) [47]	81.94	86.88	85.69
MAV + Tree [48]	68.06	60.63	77.70

**Table 3:** 5-way 1-shot and 5-way 5-shot Classification top 1 and top 5 accuracy (%) for new subject on CapgMyo with MAML. The meta batch size is set to 8. The boldface numbers represent the best classification accuracies for each dataset compared with Table 4.

Dataset	1Shot-Top1	1Shot-Top5	5Shot-Top1	5Shot-Top5
DB-a	**72.50**	**65.00**	77.50	76.49
DB-b	**62.50**	**57.50**	**83.00**	**81.00**
DB-c	**80.00**	**70.00**	**83.50**	**80.99**

**Table 4:** 5-way 1-shot and 5-way 5-shot Classification top 1 and top 5 accuracy (%) for new subject on CapgMyo with MAML. The meta batch size is set to 64. The boldface numbers represent the best classification accuracies for each dataset compared with Table 3.

Dataset	1Shot-Top1	1Shot-Top5	5Shot-Top1	5Shot-Top5
DB-a	61.56	59.06	**83.19**	**82.44**
DB-b	43.12	40.63	73.12	71.25
DB-c	68.75	65.31	82.00	80.81

**Table 5:** Comparison between previous methods and the proposed CSAC-Net in terms of the accuracy (%) of inter-subject classification on DB-a, DB-b, and DB-c (10 times average accuracy). The boldface number represents the best classification results.

Methods	DB-a	DB-b	DB-c
**STFT Spectrogram + CSAC-Net**	**82.50**	**81.00**	**80.91**
MLSVD + DL [43]	–	75.40	68.30
STFT Spectrogram + Resnet18 [45] + MAML	56.70	57.70	57.90
MAV + ED-TCN [44] + MAML	28.40	30.15	29.30
raw data + extended AdaBN [31]	–	55.30	35.10

**Table 6:** 5-way 1-shot and 5-way 5-shot classification top 1 and top 5 accuracy (%) of different input forms for new subject on CapgMyo with MAML. The meta batch size is set to 8. The boldface numbers represent the best classification accuracies for each input and dataset compared with Table 7.

input	Dataset	1Shot-Top1	1Shot-Top5	5Shot-Top1	5Shot-Top5
Raw sEMG	DB-a	32.50	30.00	**45.00**	**44.00**
	DB-b	**30.00**	27.50	**40.00**	**39.89**
	DB-c	37.50	35.00	**45.00**	**44.00**
FFT	DB-a	42.50	32.50	46.00	43.00
Specturm	DB-b	**40.00**	**37.50**	**44.50**	**43.50**
	DB-c	**42.50**	**40.00**	38.50	38.00

**Table 7:** 5-way 1-shot and 5-way 5-shot classification top 1 and top 5 accuracy (%) of different input forms for new subject on CapgMyo with MAML. The meta batch size is set to 64. The boldface numbers represent the best classification accuracies for each input and dataset compared with Table 6.

input	Dataset	1Shot-Top1	1Shot-Top5	5Shot-Top1	5Shot-Top5
Raw sEMG	DB-a	**38.13**	**37.19**	41.00	40.56
	DB-b	**30.00**	**29.06**	36.25	35.81
	DB-c	**45.00**	**43.44**	41.25	40.26
FFT	DB-a	**44.69**	**43.13**	**50.44**	**50.12**
Specturm	DB-b	32.81	31.56	40.31	39.94
	DB-c	36.25	34.38	**43.69**	**43.31**

**Table 8:** Classification validation and test accuracy (%) and time for training (ms) on new subject on CapgMyo with pretrained model by supervised learning.

Dataset	Validation Accuracy	Test Accuracy	Time for Training
DB-a	94.85	23.75	253112
DB-b	95.18	57.50	316119
DB-c	95.83	35.83	189864

**Table 9:** 5-way 1-shot and 5-way 5-shot classification training time (ms) with different meta batch size on new subject on CapgMyo by CSAC-Net with MAML.

Meta Batch Size	Dataset	1Shot	5Shot
8	DB-a	4143	5316
	DB-b	4605	5515
	DB-c	3885	5144
64	DB-a	8234	17305
	DB-b	8547	17757
	DB-c	8462	17295

**Table 10:** 5-way 1-shot and 5-way 5-shot classification with different models on new subject on CapgMyo with MAML. The meta batch size is set to 8. The boldface numbers represent the best classification accuracies for each model and dataset compared with Table 11.

Model	Dataset	1Shot-Top1	1Shot-Top5	5Shot-Top1	5Shot-Top5
SAC-Net	DB-a	**47.50**	**42.50**	71.50	67.00
	DB-b	**66.56**	**65.63**	**76.50**	**72.00**
	DB-c	**55.00**	**50.00**	**80.50**	**77.50**
CAC-Net	DB-a	**50.00**	**40.00**	63.50	62.50
	DB-b	**50.00**	**47.50**	63.00	61.50
	DB-c	**42.50**	37.50	**63.50**	61.00
CNN	DB-a	40.00	32.50	67.00	65.50
	DB-b	**52.50**	42.50	**72.50**	**71.50**
	DB-c	**47.50**	40.00	61.50	60.00

**Table 11:** 5-way 1-shot and 5-way 5-shot classification with different models on new subject on CapgMyo with MAML. The meta batch size is set to 64. The boldface numbers represent the best classification accuracies for each model and dataset compared with Table 10.

Model	Dataset	1Shot-Top1	1Shot-Top5	5Shot-Top1	5Shot-Top5
SAC-Net	DB-a	41.25	38.44	**82.75**	**81.50**
	DB-b	42.50	42.19	69.19	67.94
	DB-c	49.69	47.81	75.19	73.87
CAC-Net	DB-a	38.44	37.80	**72.94**	**72.37**
	DB-b	38.75	34.69	**63.94**	**62.75**
	DB-c	39.69	**37.81**	62.62	**62.31**
CNN	DB-a	**42.50**	**40.94**	**70.19**	**68.62**
	DB-b	44.06	**43.44**	69.25	68.75
	DB-c	43.13	**42.19**	**65.31**	**64.94**

## Data Availability

The CapgMyo [31] database is used to evaluate our method. Specific data can be found in http://zju-capg.org/research_en_electro_capgmyo.html (accessed on 29 March 2022).

## Abbreviations

The following abbreviations are used in this manuscript:

sEMG	surface electromyography
STFT	short time Fourier transform
MAML	model-agnostic meta-learning
CSAC-Net	Channel-Spatial Attention Convolution Network
FFT	fast Fourier transform
GAP	global average pooling
GWP	global max pooling
MAV	mean average value
